# Delphi survey to gather feedback on a CONSORT extension proposal for nutrition intervention trials

**DOI:** 10.1007/s00394-024-03561-1

**Published:** 2025-02-01

**Authors:** Arnold William, Carl Lachat, Sanne Ahles, Karen J. Murphy, Anne-Marie Minihane, Connie Weaver, Sangeetha Shyam, Jessica Rigutto-Farebrother, On behalf of the Federation of European Nutrition Societies (FENS) Working Groups on Improving Standards in the Science of Nutrition.

**Affiliations:** 1 Department of Food Technology, Safety and Health, Ghent University, Ghent, Belgium; 2 Department of Nutrition and Movement Sciences, Institute of Nutrition and Translational Research in Metabolism (NUTRIM), Maastricht University, Maastricht, The Netherlands; 3 BioActor BV, Maastricht, The Netherlands; 4 Clinical and Health Sciences & Alliance for Research in Exercise, Nutrition and Activity University of South Australia, P.O. Box 2471, Adelaide, SA 5001 Australia; 5 Nutrition and Preventive Medicine, Norwich Medical School, University of East Anglia (UEA), Norwich, UK; 6 Norwich Institute of Healthy Ageing, UEA, Norwich, UK; 7 School of Exercise and Nutritional Sciences, San Diego State University, San Diego, USA; 8 Centre for Translational Research, Institute for Research, Development, and Innovation (IRDI), International Medical University, Kuala Lumpur, Malaysia; 9 Departament de Bioquímica I Biotecnologia, Unitat de Nutrició Humana, Centro de Investigación Biomédica en Red de Fisiopatología de La Obesidad y Nutrición (CIBEROBN), Instituto de Salud Carlos III (ISCIII), Universitat Rovira I Virgili, Reus, Spain; 10 Laboratory of Nutrition and Metabolic Epigenetics, Institute of Food, Nutrition and Health, ETH Zürich, LFV A44, Schmelzbergstrasse 7, 8092 Zürich, Switzerland

**Keywords:** Guidelines, CONSORT, Nutrition trials, Dietary interventions, Reporting, Expert opinion

## Abstract

**Purpose:**

Inadequate reporting of nutrition data can hinder the success of nutrition health policies. CONSORT provides guidance for reporting of randomised controlled trials (RCTs) and is required by most journals today, yet reporting of nutrition interventions may benefit from a more tailored approach. A Federation of European Nutrition Societies working group was created to improve quality and completeness of reporting of nutrition trials, and our work to date features a proposal for a CONSORT extension specific to nutrition RCTs. The present manuscript describes a Delphi survey conducted to gather opinion from a wider panel of nutrition and health experts and related interest-holders on our proposal.

**Methods:**

We invited 138 potentially eligible participants to take part in the Delphi survey from a representative spread of expertise and geography. We employed a Likert scale with comments for our 32-item proposal in round 1, and a dichotomous scale with comments for our 29-item proposal in round 2. Threshold for agreement was set at ≥ 80% for both rounds.

**Results:**

Forty-seven potentially eligible participants responded to our invitation, 38 completed the first round and 36 completed the second. N = 23 (72%) items achieved ≥ 80% in round 1, and 100% of items in round 2. Three items were dropped or merged following round 1. A third Delphi round was not required to obtain consensus.

**Conclusions:**

This Delphi expert consensus proposes a 29-item checklist specific to the reporting of nutrition RCTs and will inform further development of guidance through forthcoming consensus meetings.

**Supplementary Information:**

The online version contains supplementary material available at 10.1007/s00394-024-03561-1.

## Introduction

Formulation of effective human nutrition policy for public health promotion requires sound scientific evidence generated through robust randomised controlled trials. These must be thoroughly and rigorously reported to optimally inform decision-making for public health and clinical practice in health and nutrition. Inadequate and/or non-standardized reporting of these trials can hinder the reproducibility, interpretation, and application of results.

The Consolidated Standards of Reporting Trials (CONSORT) guidelines were first published in 1996 [1], and last updated in 2010 and have strengthened reporting of trial data considerably. However, randomised controlled trials (RCTs) in nutrition may be subject to challenges in reporting when using the current CONSORT guidelines for reporting completeness [2]. Nutritional interventions can be complex, in that dietary substitution of one food for another may change not only the nutrient under study but also other nutrients that may have an effect on the host [3, 4]. Often, nutritional interventions lack a “true” (i.e., a non-intervention) control [4], and unlike pharmacological interventions, all participants have a habitual and highly heterogeneous exposure to the dietary pattern, food, or bioactive of interest, which will likely influence the response to intervention [5, 6]. Such factors require thorough examination and discussion when nutrition research is reported, a point that may not be obvious to consider when referring to the standard CONSORT guidelines. Thus, a tailored approach to reporting nutrition trials through a dedicated extension to CONSORT may be warranted.

To respond to this need, a working group was formed in 2020 as part of the Federation of European Nutrition Societies (FENS) initiative of “Improving Standards in the Science of Nutrition” [7], with the objective to improve reporting of RCTs in nutrition have previously documented our investigations into determining the need for, and proposal of, a CONSORT extension specifically for nutrition trials. Our work includes the initial drafting of a 28-item checklist addition to CONSORT by an international working group of nutrition researchers [8], gathering input from the community of nutrition researchers, scientists, practitioners, and journal editors. Moreover, we piloted our proposed CONSORT extension on several purposefully chosen published nutrition articles [2]. As part of the initiatives planned by FENS for this internationally intended extension, a Delphi survey was conducted to canvas further and galvanise final opinion on our proposal, as recommended by Moher et al. [9] in the development of reporting guidelines.

## Methods

This manuscript follows the ACcurate COnsensus Reporting Document (ACCORD) reporting guideline for consensus methods in biomedicine [10].

We published our protocol a priori as a preprint via the Open Science Framework [11]. The project was led by JRF with assistance from AW, and with the other FENS group members (CL, SA, KJM, AMM, CW, SS), as steering committee and consensus panel. All members are human nutrition scientists working in academia. In brief, following ethical clearance from the ETH Zürich, Switzerland, we invited 134 subject experts to participate in the Delphi survey. To identify potentially eligible participants, we first compiled a list of expertise as diverse as possible to ensure representation across a full range of themes and disciplines in nutrition science. Next, potentially eligible participants were identified through the authors’ wider networks, purposefully chosen for their subject matter expertise or functionality within the nutrition field (Table 1). Invitation letters together with informed consent and declaration of conflict-of-interest forms were sent by email by one author (JRF) and potentially eligible participants were given a period of one week to decide on participation. Potentially eligible participants were provided the link to our protocol, which also references our previous work upon which this survey was built.

**Table 1:** Category list for participants invited to participate in the Delphi survey^1^

Category	Examples	Number contacted	Number completing both Delphi rounds	Response rate (%)
Journal editors (ensuring international representation)	American Journal of Clinical Nutrition Journal of African Nutrition and Dietetics Nutrition and Dietetics Australia British Journal of Nutrition Nutrition Reviews Public Health Nutrition	7	4	57
Nutrition Societies (ensuring international representation)	International Union of Nutrition Societies American Society of Nutrition African Nutrition Society Nutrition Society UK Nutrition Society Australia	26	6	23
Experts in nutrition RCT methodology	Conflict of interest Ethics Data sharing RCT: feeding trials RCT: population interventions RCT: design RCT: statistics GRADE Cochrane Nutrition EQUATOR Guideline development experts	65	14	22
Professional groups	Nutritionists/ dieticians Food-based dietary guideline developers Public Health Medical community Medical writers Industry	36	12	33
		134	36	27^2^

^1^List is not exhaustive; for illustrative purposes; ^2^Percent response rate of overall number contacted. GRADE: Grading of Recommendations Assessment, Development and Evaluation; EQUATOR: Enhancing the QUAlity and Transparency Of health Research; RCT: Randomised controlled trial

Participants provided written informed consent and the conflict-of-interest declaration by return email, and following which, were sent a link to an online survey and requested to respond within two weeks, though the response period was extended to almost eight weeks due to the end of year holidays, extended leave and specific requests for extensions. The number of potential participants who declined or accepted and failed to participate was noted. After analysis of the first round and preparation of the second and final round, participants were again sent a link to an online survey and requested to respond within two weeks. Requests from participants for extensions were granted. A minimum of 20 participants were required to proceed [12].

The survey was designed based on our previous work, as outlined above. The first Delphi round consisted of Likert scale ranking questions addressing all aspects of the proposed checklist extension with corresponding open-ended questions providing the opportunity for comment on each item, including on CONSORT items for which no addition was proposed. It was piloted by 10 PhD students/scientific assistants before live release. We conducted quantitative analysis in Microsoft Excel (Washington, USA) and qualitative analyses in Microsoft Word (Washington, USA), to identify themes, suggestions, and emerging consensus. For the few missing data points (n = 12 (1%) across all items), we allocated a “neither agree nor disagree” vote. The objective of this first step was to determine agreement with the items on our proposed checklist. We calculated percent agreement according to the number of responses. The predetermined agreement rate with our proposed items was 80% at “agree” or “strongly agree”, meeting the consensus in Delphi surveys of a threshold of 70–80% of respondents indicating agreement at these levels [12]. Comments were considered and summarised qualitatively, grouped where relevant. We used the feedback from round 1 to make any edits to the proposed checklist and thereafter to develop the second Delphi survey, which also included items with agreement in round 1. Round 2 employed dichotomous (yes/no) questions to obtain agreement or disagreement on the items included within the edited checklist, with a free text comments section at the end of the survey only. Since responses to both surveys were collected individually and from participants independently, they were informed via the second-round email that the items for their review had been modified based on the findings of the first round. However, since this survey was fully anonymised, individual survey responses were not shared with the whole participant group to maintain anonymity and reduce the risk of bias from views of other participants [13]. Since, in round 2, no neutral option was available, we chose to leave the missing data points (n = 3; 0.3%) across all items) blank and calculated percent agreement according to the number of responses. Again, comments were considered and summarised qualitatively and grouped where relevant. Given the consensus, we did not proceed to a third Delphi round.

The checklists for both the first, with n = 32 items, and second, with n = 29 items, Delphi rounds are available in the Supplementary material.

## Results

The Delphi survey was conducted between 7 December 2023 and 22 January 2024 (round 1), and 12 March 2024 and 25 April 2024 (round 2). Of the 134 potentially eligible participants invited, 46 (34%) accepted, nine (7%) declined, and 79 (59%) did not respond. Participants were 61% identifying as female and 39% as male, coming from Australia, Canada, Denmark, France, India, Kenya, Malaysia, Netherlands, New Zealand, Sweden, Switzerland, Tanzania, United Kingdom, United States of America, and Zimbabwe. Self-identified areas of expertise were RCT design and conduct (n = 8, 17%), nutrition and diet studies (7, 14%), maternal and child health (3, 6%), public health nutrition (3, 6%), stable isotopes (3, 6%), nutrition epidemiology (4, 9%), micronutrients (2, 4%), personalised nutrition (2, 4%, nutrition across the lifecycle (4, 9%), sports nutrition (3, 6%), and one participant (2%) in each of the following: clinical nutrition, communications, conflict of interest and ethics, consumer science, journal editor, bioactive food components, non-communicable diseases, and food/dietary policy and regulations. Of the 46 providing written informed consent, 38 (80%) completed the first Delphi round; one participant withdrew and eight did not respond after follow-up. Of these 38, 36 participants (78% of the initial 46) completed the second round, two did not respond. The acceptance rate in each general category is given in Table 1.

In the first round, 23 (72%) items achieved an agreement of ≥ 80% (n = 5 items ≥ 95%; 4 items ≥ 90% to 95%; 6 items ≥ 85% to 90%; 8 items ≥ 80% to < 85%; Table 2).

**Table 2:** Opinions from Delphi round 1 participants on the proposed CONSORT nutrition extension checklist items

Item	Strongly agree		Agree		Neutral		Disagree		Strongly disagree	
	n	%	n	%	n	%	n	%	n	%
1a “Where possible, the type of dietary comparator should be described in the title, specifically, “RCT” for trials with a control group, “trial” where two intervention groups are used and “placebo-controlled trial” where a placebo is used as comparator”	17	44.7	16	42.1	3	7.9	2	5.3	0	0
1a “Distinguishing between an “RCT” and a “trial” is not critical”	0	0.0	4	10.5	6	15.8	28	73.7	0	0
1b “Details of the food bioactive, food/food group, dietary pattern or eating behavior intervention and comparator should be included”	23	60.5	14	36.8	1	2.6	0	0.0	0	0
1b “If nutritional status, dietary intake or eating behavior is the primary outcome should be clearly stated”	20	52.6	15	39.5	2	5.3	1	2.6	0	0
1b “Trial design, e.g., cluster, cross-over, parallel, non-inferiority should be specified”	21	55.3	14	36.8	2	5.3	1	2.6	0	0
1b “Treatment effects should be included”	16	42.1	15	39.5	6	15.8	1	2.6	0	0
1b “Whether the manuscript reports a secondary RCT analysis should be stated”	14	36.8	16	42.1	6	15.8	2	5.3	0	0
2a “The biological plausibility of the nutrition intervention and/or behavioral, physiological, or molecular mechanism underpinning the intervention impact on the primary outcome measures, should be stated”	13	34.2	16	42.1	5	13.2	4	10.5	0	0
2a “Contextualization, where relevant, to current dietary recommendations or food intake in the population of interest should be provided. The population chosen should be justified, giving details. PICO criteria should be clearly identifiable”	13	34.2	17	44.7	5	13.2	3	7.9	0	0
3a “The trial design should align with the scientific question being addressed”	25	65.8	11	28.9	1	2.6	1	2.6	0	0
3a “Duration of the trial should be appropriate for the primary and key secondary nutrition sensitive outcomes”	21	55.3	14	36.8	1	2.6	2	5.3	0	0
3a “Potential confounders should be described including baseline nutritional status (especially for the nutrient, bioactive, diet being tested to determine if participants are already adequate) and factors that could influence nutrition trial outcomes (habitual diet, socioeconomic status, season, physical activity, knowledge of participants and interventionists, especially for education interventions), carry-over effects in crossover trials”	18	47.4	13	34.2	4	10.5	3	7.9	0	0
4a “Target populations- clinical, at risk, and healthy population, specify particular dietary, physiological or nutritional characteristics targeted. List eligibility criteria related to baseline nutritional status (anthropometric, biochemical, clinical, diet, food allergies)”	23	60.5	11	28.9	1	2.6	3	7.9	0	0
5 “Dietary comparators should be well described, including details if isocaloric or not, as applicable”	21	55.3	15	39.5	1	2.6	1	2.6	0	0
5. “Details of the diet-related intervention should be given. If given, how was it prepared {form, matrix, co-ingested nutrients and constituents, food type, presentation (tablet, drink, food)}, stored, checked for bioactive constituent(s), evaluated for storage stability, and biological exposure monitored? For behavioral interventions, describe the protocol that includes how it was developed and administered and by whom and when. Description of assessment of background diets is needed as relevant”	24	63.2	9	23.7	2	5.3	3	7.9	0	0
5 “Include acceptability and tolerance of intervention”	13	34.2	16	42.1	8	21.1	1	2.6	0	0
6a “Anticipated confounders should be measured”	12	31.6	16	42.1	6	15.8	4	10.5	0	0
8a/9 “Randomization based on nutrient intake or status”	6	15.8	13	34.2	11	28.9	8	21.1	0	0
8a/9 “Allocation concealment as relevant should be described distinct from blinding”	12	31.6	19	50.0	6	15.8	1	2.6	0	0
11a “It should describe any limits to blinding and who was blinded (participants, staff who delivered the intervention, analytical staff), as well as details of concealed allocation”	19	50.0	16	42.1	3	7.9	0	0.0	0	0
12a “A priori statistical analysis plan that aligns with the study design should be described, and primary analysis should be based on intention-to-treat, with per-protocol analysis described in addition where relevant”	16	42.1	17	44.7	3	7.9	2	5.3	0	0
12a “Comparisons between intention-to-treat and per protocol analysis should be considered. Additionally, per protocol compliance cut-offs should be reported, including possible exclusion criteria for misreporting”	15	39.5	18	47.4	4	10.5	1	2.6	0	0
12a “Must adjust for stratification variables”	8	21.1	13	34.2	13	34.2	4	10.5	0	0
12b “Identify and justify data analysis choice (e.g., statistical method used to combine dietary or nutritional data, energy adjustments, intake modeling, use of weighting factors). Define stratifications and adjustments”	17	44.7	15	39.5	5	13.2	1	2.6	0	0
12b “Post SAP analysis should be clearly identified as exploratory”	12	31.6	14	36.8	11	28.9	1	2.6	0	0
21 “Generalizability with consideration to background diet and any variation in other populations, ensuring a differentiation between efficacy and effectiveness should be clearly discussed”	20	52.6	17	44.7	1	2.6	0	0.0	0	0
22 “The main findings of the paper, using intention-to-treat principles, with per protocol interpretations given in addition, depending on the objective of the study should be stated. A clear differentiation for these findings from ancillary analyses should be provided”	12	31.6	20	52.6	5	13.2	1	2.6	0	0
22 “The choice of comparator, including whether isocaloric exchange was used or not, and any bias introduced, should be discussed”	14	36.8	18	47.4	5	13.2	1	2.6	0	0
22 “Any assessment of dietary adherence should be discussed”	23	60.5	13	34.2	1	2.6	1	2.6	0	0
22 “Any relevant aspects on the active constituent of the intervention as revealed by the trial should be discussed”	16	42.1	15	39.5	4	10.5	3	7.9	0	0
22 “Any potentially false discoveries due to any adjustments used in statistical analyses should be described”	12	31.6	20	52.6	2	5.3	4	10.5	0	0
22 “Authors should distinguish clearly between statistical and clinically relevant findings, with detailed interpretation on how the findings affect clinical practice, dietary guidance, or public health recommendations, as relevant”	25	65.8	9	23.7	3	7.9	1	2.6	0	0

There was disagreement (“disagree” or “strongly disagree”) among ≥ 20% of participants for two items (1a. “distinguishing between an “RCT” and a “trial” is not critical”; and 8a/9 “randomization based on nutrient intake or status”) and ≥ 10–19% of participants for three items (6a “anticipated confounders should be measured”; 12a “must adjust for stratification variables”; 22 “any potentially false discoveries due to any adjustments used in statistical analyses should be described”). Comments on item 1a included that the RCT design should be included, as well as the investigated compound/ingredient/food. Some participants felt that “trial” is too general a term and the use of a control should be indicated as well as any specific terminology, such as “only placebo-controlled trials are RCTs”. Other participants advised the inclusion of the condition under study, as well as stipulation of details in the abstract due to title word limits. Comments on item 8a/9 were due mainly to a lack of clarity in the item description. Comments on item 6a included that it is impossible to measure known and unknown confounders, so “where possible” should be included. It was also suggested that confounding variables to be included may be identified “via use of a DAG (directed acyclic graph)”. Comments on item 12a stated that the proposed checklist should reflect trial reporting instead of design. Finally, comments on item 22 suggested that this item might introduce too many challenges in reporting, that some of the items would be better placed elsewhere, that the discussion should “detail potential limitations in interpretation due to lack or limitations in the control group (such as lack of “no treatment” control in prevention trials comparing different kinds of interventions)”, and that the “context should be included more in the reflection of the findings”.

Using the findings of round 1, we modified our proposed checklist for use in round 2. Changes can be reviewed in Table 3 and the Supplementary material.

**Table 3:** Changes to the checklist following Delphi round 1 feedback

Item in round 1	Change for round 2
1a “Where possible, the type of dietary comparator should be described in the title, specifically, “RCT” for trials with a control group, “trial” where two intervention groups are used and “placebo-controlled trial” where a placebo is used as comparator”	No change
1a “Distinguishing between an “RCT” and a “trial” is not critical”	Deleted
1b “Details of the food bioactive, food/food group, dietary pattern or eating behavior intervention and comparator should be included”	No change
1b “If nutritional status, dietary intake or eating behavior is the primary outcome should be clearly stated”	No change
1b “Trial design, e.g., cluster, cross-over, parallel, non-inferiority should be specified”	No change
1b “Treatment effects should be included”	No change
1b “Whether the manuscript reports a secondary RCT analysis should be stated”	No change
2a “The biological plausibility of the nutrition intervention and/or behavioral, physiological, or molecular mechanism underpinning the intervention impact on the primary outcome measures, should be stated”	Item moved to item 22
2a “Contextualization, where relevant, to current dietary recommendations or food intake in the population of interest should be provided. The population chosen should be justified, giving details. PICO criteria should be clearly identifiable”	PICO acronym defined
3a “The trial design should align with the scientific question being addressed”	Merged, with new text: “Describe how the trial design aligns with the scientific question being addressed and justify the duration and its appropriateness for the primary and key secondary nutrition sensitive outcomes”
3a “Duration of the trial should be appropriate for the primary and key secondary nutrition sensitive outcomes”	
3a “Potential confounders should be described including baseline nutritional status (especially for the nutrient, bioactive, diet being tested to determine if participants are already adequate) and factors that could influence nutrition trial outcomes (habitual diet, socioeconomic status, season, physical activity, knowledge of participants and interventionists, especially for education interventions), carry-over effects in crossover trials”	No change
4a “Target populations- clinical, at risk, and healthy population, specify particular dietary, physiological or nutritional characteristics targeted. List eligibility criteria related to baseline nutritional status (anthropometric, biochemical, clinical, diet, food allergies)”	No change
5 “Dietary comparators should be well described, including details if isocaloric or not, as applicable”	No change
5. “Details of the diet-related intervention should be given. If given, how was it prepared {form, matrix, co-ingested nutrients and constituents, food type, presentation (tablet, drink, food)}, stored, checked for bioactive constituent(s), evaluated for storage stability, and biological exposure monitored? For behavioral interventions, describe the protocol that includes how it was developed and administered and by whom and when. Description of assessment of background diets is needed as relevant”	Text updated to: “Details of the diet-related intervention should be given. If given, describe how it was prepared, stored, checked for bioactive constituent(s), evaluated for storage stability, and biological exposure monitored. For behavioral interventions, describe the protocol that includes how it was developed and administered and by whom and when. Description of assessment of background diets is needed as relevant”
5 “Include acceptability and tolerance of intervention”	Text updated to: “Include methods describing how acceptability and tolerance of intervention were assessed, where relevant”
6a “Anticipated confounders should be measured”	“Where possible” added to sentence end
8a/9 “Randomization based on nutrient intake or status”	Item removed
8a/9 “Allocation concealment as relevant should be described distinct from blinding”	Text updated to: “Allocation concealment should be described, as relevant and distinct from blinding”
11a “It should describe any limits to blinding and who was blinded (participants, staff who delivered the intervention, analytical staff), as well as details of concealed allocation”	No change
12a “A priori statistical analysis plan that aligns with the study design should be described, and primary analysis should be based on intention-to-treat, with per-protocol analysis described in addition where relevant”	Merged and wording changed to: “The choice for intention-to-treat or per-protocol as primary analysis should be justified, and, if relevant, comparisons between intention-to-treat and per protocol analysis should be presented (for instance, in case of high drop-out or non-compliance). Compliance cut-offs, where relevant, including possible exclusion criteria for misreporting, should be described.”
12a “Comparisons between intention-to-treat and per protocol analysis should be considered. Additionally, per protocol compliance cut-offs should be reported, including possible exclusion criteria for misreporting”	
12a “Must adjust for stratification variables”	Text updated to: “Must describe where analyses were adjusted for stratification variables.”
12b “Identify and justify data analysis choice (e.g., statistical method used to combine dietary or nutritional data, energy adjustments, intake modeling, use of weighting factors). Define stratifications and adjustments”	No change
12b “Post SAP analysis should be clearly identified as exploratory”	SAP abbreviation defined and text updated to “Statistical analyses not specified in the statistical analysis plan (SAP) should be clearly identified as exploratory”
13a to 19—no specific extension deemed necessary	18, item added: “Declare ancillary analyses as pre-specified or exploratory, reporting interaction terms, sensitivity analyses, acceptability & tolerance, and data imputation where relevant.”
21 “Generalizability with consideration to background diet and any variation in other populations, ensuring a differentiation between efficacy and effectiveness should be clearly discussed”	No change
22 “The main findings of the paper, using intention-to-treat principles, with per protocol interpretations given in addition, depending on the objective of the study should be stated. A clear differentiation for these findings from ancillary analyses should be provided”	Text updated to: “The main findings of the paper based on the objective of the study should be stated. A clear differentiation for these findings from ancillary analyses should be provided.”
22 “The choice of comparator, including whether isocaloric exchange was used or not, and any bias introduced, should be discussed”	No change
22 “Any assessment of dietary adherence should be discussed”	No change
22 “Any relevant aspects on the active constituent of the intervention as revealed by the trial should be discussed”	No change
22 “Any potentially false discoveries due to any adjustments used in statistical analyses should be described”	No change
22 “Authors should distinguish clearly between statistical and clinically relevant findings, with detailed interpretation on how the findings affect clinical practice, dietary guidance, or public health recommendations, as relevant”	No change

In the second round, all items achieved an agreement of ≥ 80% (n = 10 items ≥ 95%; 11 items ≥ 90%; 5 items ≥ 85%; 3 items ≥ 80%; Table 4). The items with the greatest disagreement by n = 7 (19%) participants were item 1b (“whether the manuscript reporting a secondary RCT analysis should be clearly stated along with the primary outcome”, and item 22 (“Any potentially false discoveries due to any adjustments used in statistical analyses should be described”), though no comments in the free text question provided justification for the disagreement.

**Table 4:** Opinions from Delphi round 2 participants on the proposed CONSORT nutrition extension checklist items

Item	Agree (Yes)		Disagree (No)	
	n	%	n	%
1a “where possible, the type of dietary comparator should be described in the title, specifically, “RCT” for trials with a control group, “trial” where two intervention groups are used and “placebo-controlled trial” where a placebo is used as comparator”	36	100.0	0	0.0
1b “details of the food bioactive, food/food group, dietary pattern or eating behavior intervention and comparator should be included”	34	94.4	2	5.6
1b “if nutritional status, dietary intake or eating behavior is the primary outcome should be clearly stated”	35	97.2	1	2.8
1b”trial design, e.g., cluster, cross-over, parallel, −1n-inferiority should be specified”	33	91.7	3	8.3
1b “treatment effects should be included”	35	97.2	1	2.8
1b “whether the manuscript reports a secondary RCT analysis should be clearly stated along with the primary outcome”	29	80.6	7	19.4
2a “contextualization, where relevant, to current dietary recommendations or food intake in the population of interest should be provided. The population chosen should be justified, giving details. PICO (Population, Intervention, Comparator, Outcome) criteria should be clearly identifiable”	30	83.3	5	13.9
3a”describe how the trial design aligns with the scientific question being addressed and justify the duration and its appropriateness for the primary and key secondary nutrition sensitive outcomes”	31	86.1	5	13.9
3a “potential confounders relevant to the scientific question should be reported, including baseline nutritional status (especially for the nutrient, bioactive, diet being tested to determine if participants are already adequate) and factors that could influence nutrition trial outcomes (habitual diet, socioeco-1mic status, season, physical activity, k-1wledge of participants and interventionists, especially for education interventions), carry-over effects in crossover trials”	33	91.7	3	8.3
4a “target populations- clinical, at risk, and healthy population, specify particular dietary, physiological or nutritional characteristics targeted. List eligibility criteria related to baseline nutritional status (anthropometric, biochemical, clinical, diet, food allergies)”	33	91.7	3	8.3
5 “dietary comparators should be well described, including details if isocaloric or −1t, as applicable”	34	94.4	2	5.6
5 “details of the diet-related intervention should be given. If given, describe how it was prepared, stored, checked for bioactive constituent(s), evaluated for storage stability, and biological exposure monitored. For behavioral interventions, describe the protocol that includes how it was developed and administered and by whom and when. Description of assessment of background diets is needed as relevant”	30	83.3	6	16.7
5 “include methods describing how acceptability and tolerance of intervention were assessed, where relevant”	33	91.7	3	8.3
56a “anticipated confounders should be described, including how they were measured, where possible”	31	86.1	5	13.9
8a/9 “allocation concealment should be described, as relevant and distinct from blinding”	34	94.4	2	5.6
11a “it should describe any limits to blinding and who was blinded (participants, staff who delivered the intervention, analytical staff), as well as details of concealed allocation”	36	100.0	0	0.0
12a “the choice for intention-to-treat or per-protocol as primary analysis should be justified, and, if relevant, comparisons between intention-to-treat and per protocol analysis should be presented (for instance, in case of high drop-out or −1n-compliance). Compliance cut-offs, where relevant, including possible exclusion criteria for misreporting, should be described”	33	91.7	3	8.3
12a “must describe where analyses were adjusted for stratification variables”	35	97.2	1	2.8
12b “identify and justify data analysis choice (e.g., statistical method used to combine dietary or nutritional data, energy adjustments, intake modeling, use of weighting factors). Define stratifications and adjustments”	34	94.4	2	5.6
12b “statistical analyses −1t specified in the statistical analysis plan (SAP) should be clearly identified as exploratory”	32	88.9	4	11.1
18 “declare ancillary analyses as pre-specified or exploratory, reporting interaction terms, sensitivity analyses, acceptability & tolerance, and data imputation where relevant”	32	88.9	4	11.1
21 “generalisability with consideration to background diet and any variation in other populations, ensuring a differentiation between efficacy and effectiveness should be clearly discussed”	34	94.4	0	0.0
22 “the main findings of the paper based on the objective of the study should be stated. A clear differentiation for these findings from ancillary analyses should be provided”	36	100.0	0	0.0
22 “the biological plausibility of the nutrition intervention and/or behavioral, physiological, or molecular mechanism underpinning the intervention impact on the primary outcome measures, should be stated”	35	97.2	1	2.8
22 “the choice of comparator, including e.g., whether isocaloric exchange was used or −1t, and any bias introduced, should be discussed”	35	97.2	1	2.8
22 “any assessment of dietary adherence should be discussed”	36	100.0	0	0.0
22 “any relevant aspects on the active constituent of the intervention as revealed by the trial should be discussed”	34	94.4	2	5.6
22 “any potentially false discoveries due to any adjustments used in statistical analyses should be described”	29	80.6	7	19.4
22 “authors should distinguish clearly between statistical and clinically relevant findings, with detailed interpretation on how the findings affect clinical practice, dietary guidance, or public health recommendations, as relevant”	34	94.4	2	5.6

Remaining comments concerned blinding: “Unlike drug trials many nutrition feeding trials cannot be blinded. This needs to be mentioned in Sect. 5 and about the description of placebo food/beverage as a comparator”; “Blinding could be problematic with dietary interventions. It should be considered in the study design but may be difficult to implement. If the primary outcome is biochemical, for example, blood level, then blinding may not be an issue”, and similarly comments recognising the challenge of dietary versus supplement interventions, which may be easier to blind. One participant was concerned about how cultural issues may be addressed in the reporting guidelines. Additional comments suggesting wording changes or rephrasing have been evaluated by the study team and in all cases adopted.

Table 5 gives the final output of this Delphi expert consensus exercise.

**Table 5:** Output of Delphi expert consensus exercise

**Section: Title and abstract**
**CONSORT checklist item 1a** • Where possible, the type of dietary comparator should be described in the title, specifically, “RCT” for trials with a control group, “trial” where two intervention groups are used and “placebo-controlled trial” where a placebo is used as comparator **CONSORT checklist item 1b** • Details of the food bioactive, food/food group, dietary pattern or eating behaviour intervention and comparator should be included • If nutritional status, dietary intake or eating behaviour is the primary outcome should be clearly stated • Trial design, e.g., cluster, cross-over, parallel, non-inferiority should be specified • Treatment effects should be included • Whether the manuscript reports a secondary RCT analysis should be clearly stated along with the primary outcome **Section: Introduction** **CONSORT checklist item 2a** • Contextualization, where relevant, to current dietary recommendations or food intake in the population of interest should be provided. The target population chosen should be justified, giving details, where possible. PICO (Population, Intervention, Comparator, Outcome) criteria should be clearly identifiable **CONSORT checklist item 2b.** This recommendation is considered sufficient for nutrition trials. No specific extension is required **Section: Methods** ***Trial Design*** **CONSORT checklist item 3a** • Describe how the trial design aligns with the scientific question being addressed and justify the duration and its appropriateness for the primary and key secondary nutrition sensitive outcomes • Potential confounders relevant to the scientific question should be reported, including baseline nutritional status (especially for the nutrient, bioactive, diet being tested to determine if participants are already adequate) and factors that could influence nutrition trial outcomes (for example, habitual diet, socioeconomic status, season, physical activity, knowledge of participants and interventionists, especially for education interventions), carry-over effects in crossover trials **CONSORT checklist item 3b.** This recommendation is considered sufficient for nutrition trials. No specific extension is required ***Participants*** **CONSORT checklist item 4a** • Target populations- clinical, at risk, and healthy population, specify particular dietary, physiological or nutritional characteristics targeted. List eligibility criteria related to baseline nutritional status (anthropometric, biochemical, clinical, diet, food allergies) **CONSORT checklist item 4b.** This recommendation is considered sufficient for nutrition trials. No specific extension is required ***Interventions*** **CONSORT checklist item 5** • Dietary comparators should be well described, including details if isocaloric or not, as applicable • Details of the diet-related intervention should be given. If given, describe how it was prepared, stored, checked for bioactive constituent(s), evaluated for storage stability, and biological exposure monitored. For behavioural interventions, describe the protocol that includes how it was developed and administered and by whom and when. Description of assessment of background diets is needed as relevant • Include methods describing how acceptability and tolerance of intervention were assessed, where relevant ***Outcomes*** **CONSORT checklist item 6a** • Anticipated confounders should be described, including how they were measured, where possible **CONSORT checklist item 6b.** This recommendation is considered sufficient for nutrition trials. No specific extension is required ***Sample size*** **CONSORT checklist items 7a and 7b.** These recommendations are considered sufficient for nutrition trials. No specific extension is required ***Randomization: sequence generation, allocation concealment and implementation*** **CONSORT checklist items 8a and 9** • Allocation concealment should be described, as relevant and distinct from blinding **CONSORT checklist items 8b and 10.** These recommendations are considered sufficient for nutrition trials. No specific extension is required ***Blinding*** **CONSORT checklist item 11a** • Describe any limits to blinding and who was blinded (participants, staff who delivered the intervention, analytical staff), as well as details of concealed allocation C**ONSORT checklist item 11b.** This recommendation is considered sufficient for nutrition trials. No specific extension is required ***Statistical methods*** **CONSORT checklist item 12a** • The choice for intention-to-treat or per-protocol as primary analysis should be justified, and, if relevant, comparisons between intention-to-treat and per protocol analysis should be presented (for instance, in case of high drop-out or non-compliance). Compliance cut-offs, where relevant, including possible exclusion criteria for misreporting, should be described • Must describe where analyses were adjusted for stratification variables **CONSORT checklist item 12b** • Identify and justify data analysis choice (e.g., statistical method used to combine dietary or nutritional data, energy adjustments, intake modelling, use of weighting factors). Define stratifications and adjustments • Statistical analyses not specified in the statistical analysis plan (SAP) should be clearly identified as exploratory

**Section: Results**
**CONSORT checklist items 13a to 17.** These recommendations are considered sufficient for nutrition trials. No specific extension is required **CONSORT checklist item 18** • Declare ancillary analyses as pre-specified or exploratory, reporting interaction terms, sensitivity analyses, acceptability & tolerance, and data imputation where relevant **Section: Discussion** ***Limitations*** **CONSORT checklist item 20.** This recommendation is considered sufficient for nutrition trials. No specific extension is required ***Generalisability*** **CONSORT checklist item 21** • Generalisability with consideration to background diet and any variation in other populations, ensuring a differentiation between efficacy and effectiveness should be clearly discussed ***Interpretation*** **CONSORT checklist item 22** • The main findings of the paper based on the objective of the study should be stated. A clear differentiation for these findings from ancillary analyses should be provided • The biological plausibility of the nutrition intervention and/or behavioural, physiological, or molecular mechanism underpinning the intervention impact on the primary outcome measures, should be stated • The choice of comparator, including e.g., whether isocaloric exchange was used or not, and any bias introduced, should be discussed • Any assessment of dietary adherence should be discussed • Any relevant aspects on the active constituent of the intervention as revealed by the trial should be discussed • Any potentially false discoveries due to any adjustments used in statistical analyses should be described • Authors should distinguish clearly between statistical and clinically relevant findings, with detailed interpretation on how the findings affect clinical practice, dietary guidance, or public health recommendations, as relevant **Section: Other information** **CONSORT checklist items 23 to 26.** These recommendations are considered sufficient for nutrition trials. No specific extension is required

## Discussion

Delphi surveys are an invaluable method for gathering data by engaging a panel of experts to contribute their opinions and make judgements on specific issues to reach a group consensus. The technique enables researchers to interact simultaneously with a number of interest-holders in a given field who are experienced and trained in their specialised area of knowledge, thus permitting a deeper understanding of real-world opinions [14]. Being both quantitative and qualitative, Delphi surveys allow researchers to deal systematically with complex issues for discussion, for which an expert level of knowledge and experience is usually necessary [15]. Moreover, the Delphi technique is also flexible as it allows disagreements between participants yet can reach a consensus decision without causing conflict. Since Delphi Surveys are conducted anonymously between participants, the risk of influence by more dominant participants is reduced [16].

Typical Delphi survey methods include a first, qualitative step with open-ended questions to facilitate the collection of as much feedback as possible and to avoid bias from the research team [13]. The second and any further steps may be more quantitative, as agreement is sought. Although the Delphi process can continue endlessly until there is agreement between all participants, normally a maximum of three rounds suffice to reach consensus [17].

However, there are important limitations to a Delphi survey. First of all, by their nature, Delphi surveys require people to respond. We approached 134 interest-holders to contribute yet had a response rate to invitations of n = 46 (34%), with 38 and 36 respondents completing the first and second Delphi rounds, respectively. Inadvertent recruitment bias could have contributed to this number; we asked potentially eligible participants to respond within one week to our invitation, which may have deterred responses or caused confusion with these persons mistakenly thinking that the survey would also require a response within the same week. Attrition is an important issue in Delphi surveys, since attrition of participants may cause overestimation of the degree of consensus reached in the final round [18]. Attrition rates may range from 0 to 92% in classical Delphi surveys [19]. Yet, we lost only two respondents between rounds 1 and 2, which may indicate the commitment to better reporting of nutrition trials by those who did respond. The general low response rates to our invitations could have been due to the proximity to the end of year holidays or may indicate survey fatigue [20–22]. In surveys, respondents generally only react to what is being proposed, and though free text fields may be provided, as in our survey, completion of those fields may be biassed by what has been presented, preventing respondents from generating new ideas and causing blind spots [13]. Respondents in a Delphi survey should ideally come from a representative background within the area of study. Through our initial list, we sought to achieve this, both through subject expertise and geography. Though we have a wide representativity in expertise in our final respondents, as described previously, missing specialties include biostatistics, Cochrane, consumer behaviour, data sharing, feeding trials, food-based dietary guideline development, GRADE, health economics, the industry, and representatives from journals and nutrition societies (Table 1). Further, when reviewing the final list of respondents, we have likely recruited primarily native English speakers or those who are fluent in English. Given that we reached out to experts who actively publish in the scientific press, language may not have been a barrier, however, English proficiency may have influenced both the response rate and the responses. Our final response rate also limits geographical representativity, and interest-holders from South America and Africa are under-represented.

Our invitation group was primarily made up of established professionals with expertise in the conduct of RCTs in nutrition (n = 65; Table 1). Purposeful sampling is usually employed in qualitative research with the advantage of gathering comments and opinions based on experience [23]. However, subjective purposeful sampling may increase a risk for bias in respondents compared to subjects enrolled via an open call [24]. Nevertheless, this group had the lowest response rate at 22%. Representatives from nutrition societies also had a low response rate of 23%. This is disappointing, since the global nutrition societies represent a valuable route of dissemination for the final CONSORT nutrition recommendations that will be proposed. More positively, four of the seven (57%) journal editors invited completed both Delphi rounds, possibly indicating a strong interest and perception of added value of this initiative for quality publications.

Furthermore, we missed an important interest-holder group: early career researchers. Established researchers are, theoretically, more likely to be familiar with what is expected from proper reporting of trials in nutrition, and perhaps themselves have been faced with challenges when reporting has been suboptimal, e.g. in systematic reviews or when planning new studies based on findings in the literature. Early career researchers are less likely to have this level of experience and are therefore more likely to need to rely on reporting guidelines whilst preparing their manuscripts. To counter this, we aim to pilot the final product of this process with early-career researchers to ensure that the product is unambiguous and user-friendly.

The output of this Delphi consensus exercise is a refined proposal of 29 statements specific to nutrition. As described above, this is the product of several work streams by the FENS working group [2, 8, 11, 25], about which the nutrition community has been generally very positive. Indeed, comments obtained through this Delphi exercise have been unequivocal; we did not receive any indication that additional guidance for nutrition trials is not necessary. This could be related to sampling and the points outlined above; however, the process is not yet complete, and the authors invite the reader to reach out with any concerns or further points that should be considered.

Comments from respondents were helpful in several ways, in particular by helping to focus statements on reporting of nutrition trials, not their methodology. This challenge was already evoked in our second manuscript [2], and, as is often the case, is most effectively highlighted by those who are not directly involved in proposal drafting.

The outcome of this Delphi exercise provides expert recommendations that will feed into formalising additional guidance for reporting nutrition trials. This process and the final guidance will not only promote full and standardised reporting of nutrition trials, but also help with study design and in training research students. We have focused our efforts on the derivation of a checklist, following similar examples such as the development of the STrengthening the Reporting of OBservational studies in Epidemiology (STROBE) extension for nutrition studies, STROBE-nut [26], however, the final product of this effort could also take the form of written guidance, a statement paper or opinion piece. Further, there will be a need to align the final product with the forthcoming update of the CONSORT statement concerning both content and timing, for which the team are closely working with the EQUATOR network and authors of the CONSORT statement update.

Key findings of our Delphi survey were the high level of agreement achieved by round 2, with 21 items reaching a ≥ 90% agreement, and two items (1b: “whether the manuscript reporting a secondary RCT analysis should be clearly stated along with the primary outcome”; and 22: “any potentially false discoveries due to any adjustments used in statistical analyses should be described”) at 19% disagreement. Though comment was not provided to elaborate on this disagreement, it should be considered as the final product is derived. Further, unresolved comments including those around blinding, and the interesting point of culture as a relevant factor in nutrition RCTs, in particular, reporting of cultural mechanisms around primary outcomes and ancillary analyses, deserve discussion.

Given the methodological complementarity of both groups, a collaboration was formed in 2023 between the FENS working group and the Supporting Transparency And Reproducibility in studies of NUTritional interventions (STAR-NUT) working group hosted within EQUATOR [25]. To address the issues raised by our Delphi survey and bring the ensemble of the learning by FENS and STAR-nut together with the responses and comments canvassed from the nutrition community and peer review of this manuscript, a consensus meeting will be held between the FENS and STAR-nut working groups and other key interest-holders. All material will be presented and considered at this meeting to decide on and draft a final product that will support and improve the rigour in reporting of RCTs in human nutrition and nutrition science.

## Supplementary Information

Below is the link to the electronic supplementary material.

Supplementary file1 (PDF 879 KB)
