# Supplementary material for: Delphi survey to gather feedback on a CONSORT extension proposal for nutrition intervention trials
Source: Eur J Nutr. 2025 Feb 1;64(2):76. doi: 10.1007/s00394-024-03561-1 (PMC11787169; doi:10.1007/s00394-024-03561-1)
Supplement: Supplementary file 1 — Supplementary file1 (PDF 879 KB) [file 394_2024_3561_MOESM1_ESM.pdf]

Delphi Survey to Gather Feedback on a CONSORT Extension Proposal for Nutrition Intervention Trials

Supplementary Material 1

The following pages provide the Delphi round 1 and 2 survey text, and are also available at https://doi.org/10.31219/osf.io/ukwhb:

Rigutto-Farebrother J, William A, Ahles S, Murphy K, Shyam S, Minihane AM, Weaver C, Lachat C (2023) Protocol: A Delphi Survey to Gather Feedback on a CONSORT Extension Proposal for Nutrition Intervention Trials.

Delphi round I

CONSORT extension for nutrition trials

Section: Title and abstract

CONSORT checklist item 1a. The current CONSORT guideline stipulates that a study should be identified as a randomized trial in the title. Please indicate your agreement with the following two statements about an addition to this stipulation for nutrition RCTs:

	Strongly Disagree	Disagree	Neither agree nor disagree	Agree	Strongly Agree
Where possible, the type of dietary comparator should be described in the title, specifically, “RCT” for trials with a control group, “trial” where two intervention groups are used and “placebo-controlled trial” where a placebo is used as comparator	☐	☐	☐	☐	☐

Distinguishing between an “RCT” and a “trial” is not critical	☐	☐	☐	☐	☐

CONSORT checklist item 1b. The current CONSORT guideline stipulates a structured summary of trial design, methods, results, and conclusions. For the following statements as proposed additions for nutrition RCTs in the title and abstract of reports, please indicate your agreement:

	**Strongly Disagree**	**Disagree**	**Neither agree nor disagree**	**Agree**	**Strongly Agree**
Details of the food bioactive, food/food group, dietary pattern or eating behavior intervention *and comparator* should be included	☐	☐	☐	☐	☐
If nutritional status, dietary intake or eating behavior is the primary outcome should be clearly stated	☐	☐	☐	☐	☐
Trial design, e.g., cluster, cross-over, parallel, non-inferiority should be specified	☐	☐	☐	☐	☐
Treatment effects should be included	☐	☐	☐	☐	☐
Whether the manuscript reports a secondary RCT analysis should be stated	☐	☐	☐	☐	☐

If you wish to provide alternative wording suggestions or have other issues to raise about these CONSORT checklist items, please comment below.

Section: Introduction

CONSORT checklist item 2a. The current CONSORT guideline stipulates that a description of scientific background and rationale for the study should be given. Please indicate your agreement with the following two statements about an addition to this stipulation for nutrition RCTs:

	**Strongly Disagree**	**Disagree**	**Neither agree nor disagree**	**Agree**	**Strongly Agree**
The biological plausibility of the nutrition intervention and/or behavioral, physiological, or molecular mechanism underpinning the intervention impact on the primary outcome measures, should be stated	☐	☐	☐	☐	☐
Contextualization, where relevant, to current dietary recommendations or food intake in the population of interest should be provided. The population chosen should be justified, giving details. PICO criteria should be clearly identifiable.	☐	☐	☐	☐	☐

CONSORT checklist item 2b. This recommendation is considered sufficient for nutrition trials. No specific extension is required.

If you wish to provide alternative wording suggestions or have other issues to raise about this CONSORT checklist items, please comment below.

Section: Methods

Trial Design

CONSORT checklist item 3a. The current CONSORT guideline stipulates that there should be a clear description of trial design included in the methods. Please indicate your agreement with the following statements about an addition to this stipulation for nutrition RCTs:

	Strongly Disagree	Disagree	Neither agree nor disagree	Agree	Strongly Agree
The trial design should align with the scientific question being addressed	☐	☐	☐	☐	☐
Duration of the trial should be appropriate for the primary and key secondary nutrition sensitive outcomes	☐	☐	☐	☐	☐
Potential confounders should be described including baseline nutritional status (especially for the nutrient, bioactive, diet being tested to determine if participants are already adequate) and factors that could influence nutrition trial outcomes (habitual diet, socioeconomic status, season, physical activity, knowledge of participants and interventionists, especially for education interventions), carry-over effects in crossover trials	☐	☐	☐	☐	☐

CONSORT checklist item 3b. This recommendation is considered sufficient for nutrition trials. No specific extension is required.

If you wish to provide alternative wording suggestions or have other issues to raise about this CONSORT checklist item, please comment below.

Participants

CONSORT checklist item 4a. The current CONSORT guideline stipulates participants eligibility criteria should be described Please indicate your agreement with the following statement about an addition to this stipulation for nutrition RCTs:

	Strongly Disagree	Disagree	Neither agree nor disagree	Agree	Strongly Agree
Target populations- clinical, at risk, and healthy population, specify particular dietary, physiological or nutritional characteristics targeted. List eligibility criteria related to baseline nutritional status (anthropometric, biochemical, clinical, diet, food allergies).	☐	☐	☐	☐	☐

CONSORT checklist item 4b. This recommendation is considered sufficient for nutrition trials. No specific extension is required.

If you wish to provide alternative wording suggestions or have other issues to raise about this CONSORT checklist item, please comment below.

Interventions

CONSORT checklist item 5. The current CONSORT guideline stipulates reporting interventions for each group with sufficient details to allow replication, including how and when they were actually administered. Please indicate your agreement with the following statements about an addition to this stipulation for nutrition RCTs:

	Strongly Disagree	Disagree	Neither agree nor disagree	Agree	Strongly Agree
Dietary comparators should be well described, including details if isocaloric or not, as applicable	☐	☐	☐	☐	☐

Details of the diet-related intervention should be given. If given, how was it prepared {form, matrix, co-ingested nutrients and constituents, food type, presentation (tablet, drink, food)}, stored, checked for bioactive constituent(s), evaluated for storage stability, and biological exposure monitored? For behavioral interventions, describe the protocol that includes how it was developed and administered and by whom and when. Description of assessment of background diets is needed as relevant	☐	☐	☐	☐	☐
Include acceptability and tolerance of intervention	☐	☐	☐	☐	☐

Outcomes

CONSORT checklist item 6a. The current CONSORT guideline stipulates defining completely pre-specified primary and secondary outcome measures, including how and when they were assessed. Please indicate your agreement with the following statement about an addition to this stipulation for nutrition RCTs:

	Strongly Disagree	Disagree	Neither agree nor disagree	Agree	Strongly Agree
Anticipated confounders should be measured	☐	☐	☐	☐	☐

CONSORT checklist item 6b. This recommendation is considered sufficient for nutrition trials. No specific extension is required.

If you wish to provide alternative wording suggestions or have other issues to raise about this CONSORT checklist item, please comment below.

Sample size

CONSORT checklist items 7a and 7b. These recommendations are considered sufficient for nutrition trials. No specific extension is required.

If you wish to provide alternative wording suggestions or have other issues to raise about these CONSORT checklist items, please comment below.

Randomization: sequence generation, allocation concealment and implementation

CONSORT checklist items 8a and 9. The current CONSORT guideline stipulates that randomization should be well described showing the methods used to generate the random allocation sequence, mechanisms used to implement the random allocation sequence and steps taken to conceal the sequence until interventions were assigned should be described. Please indicate your agreement with the following statement about an addition to this stipulation for nutrition RCTs:

	**Strongly Disagree**	**Disagree**	**Neither agree nor disagree**	**Agree**	**Strongly Agree**
Randomization based on nutrient intake or status	☐	☐	☐	☐	☐
Allocation concealment as relevant should be described distinct from blinding	☐	☐	☐	☐	☐

CONSORT checklist items 8b and 10. These recommendations are considered sufficient for nutrition trials. No specific extension is required.

If you wish to provide alternative wording suggestions or have other issues to raise about this CONSORT checklist item, please comment below.

Blinding

CONSORT checklist item 11a. The current CONSORT guideline stipulates reporting that, if blinding was done, who was blinded after assignment to interventions and how this was achieved. Please indicate your agreement with the following statement about an addition to this stipulation for nutrition RCTs:

	**Strongly Disagree**	**Disagree**	**Neither agree nor disagree**	**Agree**	**Strongly Agree**
It should describe any limits to blinding and who was blinded (participants, staff who delivered the intervention, analytical staff), as well as details of concealed allocation	☐	☐	☐	☐	☐

CONSORT checklist item 11 b. This recommendation is considered sufficient for nutrition trials. No specific extension is required.

If you wish to provide alternative wording suggestions or have other issues to raise about this CONSORT checklist item, please comment below.

Statistical methods

CONSORT checklist item 12a. The current CONSORT guideline stipulates that statistical methods used to compare groups for primary and secondary outcomes and methods for additional analyses, such as subgroup analyses and adjusted analyses, should be described. Please indicate your agreement with the following statement about an addition to this stipulation for nutrition RCTs:

	**Strongly Disagree**	**Disagree**	**Neither agree nor disagree**	**Agree**	**Strongly Agree**

A priori statistical analysis plan that aligns with the study design should be described, and primary analysis should be based on intention-to-treat, with per-protocol analysis described in addition where relevant.	☐	☐	☐	☐	☐
Comparisons between intention-to-treat and per protocol analysis should be considered. Additionally, per protocol compliance cut-offs should be reported, including possible exclusion criteria for misreporting.	☐	☐	☐	☐	☐
Must adjust for stratification variables	☐	☐	☐	☐	☐

CONSORT checklist item 12b. The current CONSORT guideline stipulates that statistical methods used to compare groups for primary and secondary outcomes and methods for additional analyses, such as subgroup analyses and adjusted analyses, should be described. Please indicate your agreement with the following statement about an addition to this stipulation for nutrition RCTs:

	**Strongly Disagree**	**Disagree**	**Neither agree nor disagree**	**Agree**	**Strongly Agree**
Identify and justify data analysis choice (e.g., statistical method used to combine dietary or nutritional data, energy adjustments, intake modeling, use of weighting factors). Define stratifications and adjustments.	☐	☐	☐	☐	☐
Post SAP analysis should be clearly identified as exploratory	☐	☐	☐	☐	☐

If you wish to provide alternative wording suggestions or have other issues to raise about these CONSORT checklist items, please comment below. Please ensure to note the checklist item relating to your comment.

Section: Results

CONSORT checklist items 13a to 19. These recommendations are considered sufficient for nutrition trials. No specific extension is required.

If you wish to provide alternative wording suggestions or have other issues to raise about these CONSORT checklist items, please comment below. Please ensure to note the checklist item relating to your comment.

Section: Discussion

Limitations

CONSORT checklist item 20. This recommendation is considered sufficient for nutrition trials. No specific extension is required.

If you wish to provide alternative wording suggestions or have other issues to raise about this CONSORT checklist item, please comment below.

Generalizability

CONSORT checklist item 21. The current CONSORT guideline stipulates that a discussion of the generalizability (external validity, applicability) of the trial findings should be provided. Please indicate your agreement with the following statement about an addition to this stipulation for nutrition RCTs:

	**Strongly Disagree**	**Disagree**	**Neither agree**	**Agree**	**Strongly Agree**

			nor disagree		
Generalizability with consideration to background diet and any variation in other populations, ensuring a differentiation between efficacy and effectiveness should be clearly discussed.	☐	☐	☐	☐	☐

Interpretation

CONSORT checklist item 22. The current CONSORT guideline stipulates that statistical methods used to compare groups for primary and secondary outcomes and methods for additional analyses, such as subgroup analyses and adjusted analyses, should be described. Please indicate your agreement with the following statement about an addition to this stipulation for nutrition RCTs:

	**Strongly Disagree**	**Disagree**	**Neither agree nor disagree**	**Agree**	**Strongly Agree**
The main findings of the paper, using intention-to-treat principles, with per protocol interpretations given in addition, depending on the objective of the study should be stated. A clear differentiation for these findings from ancillary analyses should be provided.	☐	☐	☐	☐	☐
The choice of comparator, including whether isocaloric exchange was used or not, and any bias introduced, should be discussed.	☐	☐	☐	☐	☐
Any assessment of dietary adherence should be discussed.	☐	☐	☐	☐	☐

Any relevant aspects on the active constituent of the intervention as revealed by the trial should be discussed.	☐	☐	☐	☐	☐
Any potentially false discoveries due to any adjustments used in statistical analyses should be described.	☐	☐	☐	☐	☐
Authors should distinguish clearly between statistical and clinically relevant findings, with detailed interpretation on how the findings affect clinical practice, dietary guidance, or public health recommendations, as relevant.	☐	☐	☐	☐	☐

If you wish to provide alternative wording suggestions or have other issues to raise about these CONSORT checklist items, please comment below. Please ensure to note the checklist item relating to your comment.

Section: Other information

CONSORT checklist items 23 to 26. These recommendations are considered sufficient for nutrition trials. No specific extension is required.

If you wish to provide alternative wording suggestions or have other issues to raise about these CONSORT checklist items, please comment below. Please ensure to note the checklist item relating to your comment.

Delphi round II

Section: Title and abstract

CONSORT checklist item 1a. The current CONSORT guideline stipulates that a study should be identified as a randomized trial in the title. Please indicate your agreement with the following statement about an addition to this stipulation for nutrition RCTs:

	**Yes**	**No**
Where possible, the type of dietary comparator should be described in the title, specifically, “RCT” for trials with a control group, “trial” where two intervention groups are used and “placebo-controlled trial” where a placebo is used as comparator	☐	☐

CONSORT checklist item 1b. The current CONSORT guideline stipulates a structured summary (abstract) of trial design, methods, results, and conclusions. For the following statements as proposed additions for nutrition RCTs in the abstract of reports, please indicate your agreement:

	**Yes**	**No**
Details of the food bioactive, food/food group, dietary pattern or eating behavior intervention *and comparator* should be included	☐	☐
If nutritional status, dietary intake or eating behavior is the primary outcome should be clearly stated	☐	☐
Trial design, e.g., cluster, cross-over, parallel, non-inferiority should be specified	☐	☐
Treatment effects should be included	☐	☐
Whether the manuscript reports a secondary RCT analysis should be clearly stated along with the primary outcome	☐	☐

Section: Introduction

CONSORT checklist item 2a. The current CONSORT guideline stipulates that a description of scientific background and rationale for the study should be given. Please indicate your agreement with the following two statements about an addition to this stipulation for nutrition RCTs:

	Yes	No
Contextualization, where relevant, to current dietary recommendations or food intake in the population of interest should be provided. The population chosen should be justified, giving details. PICO (Population, Intervention, Comparator, Outcome) criteria should be clearly identifiable.	☐	☐

CONSORT checklist item 2b. This recommendation is considered sufficient for nutrition trials. No specific extension is required.

Section: Methods

Trial Design

CONSORT checklist item 3a. The current CONSORT guideline stipulates that there should be a clear description of trial design included in the methods. Please indicate your agreement with the following statements about an addition to this stipulation for nutrition RCTs:

	Yes	No
Describe how the trial design aligns with the scientific question being addressed and justify the duration and its appropriateness for the primary and key secondary nutrition sensitive outcomes	☐	☐
Potential confounders relevant to the scientific question should be reported, including baseline nutritional status (especially for the nutrient, bioactive, diet being tested to determine if participants are already adequate) and factors that could influence nutrition trial outcomes (habitual diet, socioeconomic status, season, physical activity, knowledge of participants and interventionists, especially for education interventions), carry-over effects in crossover trials	☐	☐

CONSORT checklist item 3b. This recommendation is considered sufficient for nutrition trials. No specific extension is required.

Participants

CONSORT checklist item 4a. The current CONSORT guideline stipulates participants eligibility criteria should be described Please indicate your agreement with the following statement about an addition to this stipulation for nutrition RCTs:

	Yes	No
Target populations- clinical, at risk, and healthy population, specify particular dietary, physiological or nutritional characteristics targeted. List eligibility criteria related to baseline nutritional status (anthropometric, biochemical, clinical, diet, food allergies).	☐	☐

CONSORT checklist item 4b. This recommendation is considered sufficient for nutrition trials. No specific extension is required.

Interventions

CONSORT checklist item 5. The current CONSORT guideline stipulates reporting interventions for each group with sufficient details to allow replication, including how and when they were actually administered. Please indicate your agreement with the following statements about an addition to this stipulation for nutrition RCTs:

	Yes	No
Dietary comparators should be well described, including details if isocaloric or not, as applicable	☐	☐
Details of the diet-related intervention should be given. If given, describe how it was prepared, stored, checked for bioactive constituent(s), evaluated for storage stability, and biological exposure monitored. For behavioral interventions, describe the protocol that includes how it was developed and administered and by whom and when. Description of assessment of background diets is needed as relevant.	☐	☐
Include methods describing how acceptability and tolerance of intervention were assessed, where relevant	☐	☐

Outcomes

CONSORT checklist item 6a. The current CONSORT guideline stipulates defining completely pre-specified primary and secondary outcome measures, including how and when they were assessed. Please indicate your agreement with the following statement about an addition to this stipulation for nutrition RCTs:

	Yes	No
Anticipated confounders should be described, including how they were measured, where possible	☐	☐

CONSORT checklist item 6b. This recommendation is considered sufficient for nutrition trials. No specific extension is required.

Sample size

CONSORT checklist items 7a and 7b. These recommendations are considered sufficient for nutrition trials. No specific extension is required.

Randomization: sequence generation, allocation concealment and implementation

CONSORT checklist items 8a and 9. The current CONSORT guideline stipulates that randomization should be well described showing the methods used to generate the random allocation sequence, mechanisms used to implement the random allocation sequence and steps taken to conceal the sequence until interventions were assigned should be described. Please indicate your agreement with the following statement about an addition to this stipulation for nutrition RCTs:

	Yes	No
Allocation concealment should be described, as relevant and distinct from blinding	☐	☐

CONSORT checklist items 8b and 10. These recommendations are considered sufficient for nutrition trials. No specific extension is required.

Blinding

CONSORT checklist item 11a. The current CONSORT guideline stipulates reporting that, if blinding was done, who was blinded after assignment to interventions and how this was achieved. Please indicate your agreement with the following statement about an addition to this stipulation for nutrition RCTs:

	Yes	No
It should describe any limits to blinding and who was blinded (participants, staff who delivered the intervention, analytical staff), as well as details of concealed allocation	☐	☐

CONSORT checklist item 11 b. This recommendation is considered sufficient for nutrition trials. No specific extension is required.

Statistical methods

CONSORT checklist item 12a. The current CONSORT guideline stipulates that statistical methods used to compare groups for primary and secondary outcomes and methods for additional analyses, such as subgroup analyses and adjusted analyses, should be described. Please indicate your agreement with the following statement about an addition to this stipulation for nutrition RCTs:

	**Yes**	**No**
The choice for intention-to-treat or per-protocol as primary analysis should be justified, and, if relevant, comparisons between intention-to-treat and per protocol analysis should be presented (for instance, in case of high drop-out or non-compliance). Compliance cut-offs, where relevant, including possible exclusion criteria for misreporting, should be described.	☐	☐
Must describe where analyses were adjusted for stratification variables.	☐	☐

CONSORT checklist item 12b. The current CONSORT guideline stipulates that statistical methods used to compare groups for primary and secondary outcomes and methods for additional analyses, such as subgroup analyses and adjusted analyses, should be described. Please indicate your agreement with the following statement about an addition to this stipulation for nutrition RCTs:

	**Yes**	**No**
Identify and justify data analysis choice (e.g., statistical method used to combine dietary or nutritional data, energy adjustments, intake modeling, use of weighting factors). Define stratifications and adjustments.	☐	☐
Statistical analyses not specified in the statistical analysis plan (SAP) should be clearly identified as exploratory	☐	☐

Section: Results

CONSORT checklist items 13a to 17. These recommendations are considered sufficient for nutrition trials. No specific extension is required.

CONSORT checklist item 18: The current CONSORT guideline stipulates that ancillary analyses should be declared as pre-specified or exploratory, with interaction terms,

sensitivity analyses and data imputation being reported, where relevant. Please indicate your agreement with the following statement about an addition to this stipulation for nutrition RCTs:

	Yes	No
Declare ancillary analyses as pre-specified or exploratory, reporting interaction terms, sensitivity analyses, acceptability & tolerance, and data imputation where relevant.	☐	☐

Section: Discussion

Limitations

CONSORT checklist item 20. This recommendation is considered sufficient for nutrition trials. No specific extension is required.

Generalizability

CONSORT checklist item 21. The current CONSORT guideline stipulates that a discussion of the generalizability (external validity, applicability) of the trial findings should be provided. Please indicate your agreement with the following statement about an addition to this stipulation for nutrition RCTs:

	Yes	No
Generalizability with consideration to background diet and any variation in other populations, ensuring a differentiation between efficacy and effectiveness should be clearly discussed.	☐	☐

Interpretation

CONSORT checklist item 22. The current CONSORT guideline stipulates that statistical methods used to compare groups for primary and secondary outcomes and methods for additional analyses, such as subgroup analyses and adjusted analyses, should be described. Please indicate your agreement with the following statement about an addition to this stipulation for nutrition RCTs:

	Yes	No

The main findings of the paper based on the objective of the study should be stated. A clear differentiation for these findings from ancillary analyses should be provided.	☐	☐
The biological plausibility of the nutrition intervention and/or behavioral, physiological, or molecular mechanism underpinning the intervention impact on the primary outcome measures, should be stated.	☐	☐
The choice of comparator, including e.g., whether isocaloric exchange was used or not, and any bias introduced, should be discussed.	☐	☐
Any assessment of dietary adherence should be discussed.	☐	☐
Any relevant aspects on the active constituent of the intervention as revealed by the trial should be discussed.	☐	☐
Any potentially false discoveries due to any adjustments used in statistical analyses should be described.	☐	☐
Authors should distinguish clearly between statistical and clinically relevant findings, with detailed interpretation on how the findings affect clinical practice, dietary guidance, or public health recommendations, as relevant.	☐	☐

Section: Other information

CONSORT checklist items 23 to 26. These recommendations are considered sufficient for nutrition trials. No specific extension is required.

If you wish to provide alternative wording suggestions for the proposed items, or have other issues to raise about the CONSORT checklist as it applies to nutrition RCTs, please use the box below to comment, clearly indicating the checklist item number to which you are referring.

End of survey.

Supplementary Material 2

Figures S1 and S2 visually describe the results of Delphi rounds 1 and 2, respectively.

Figure S1. Range of agreement and disagreement with our proposed items in the Delphi survey round 1

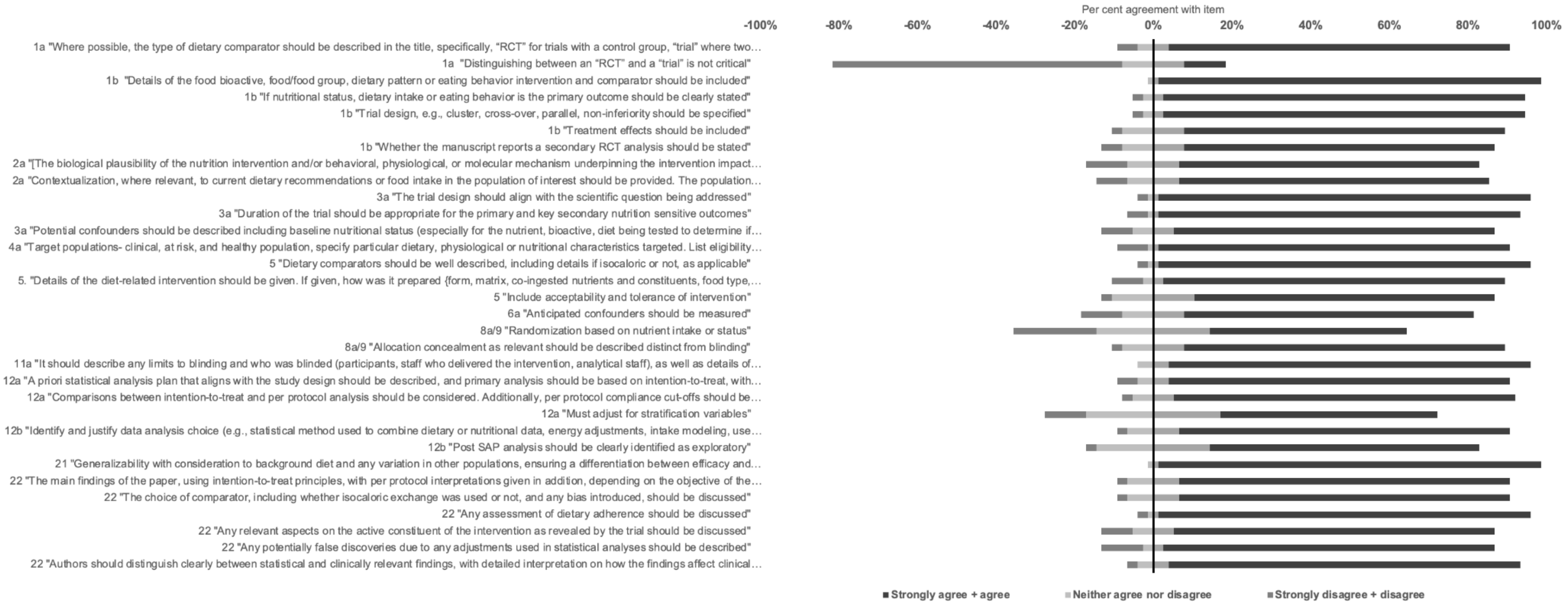

Figure legend: Per our protocol, strongly agree and agree, and strongly disagree and disagree, were combined. Agreement with the statement had to be >80% for transferral to Delphi round 2. Some items are truncated due to length; the full text for each item can be found in the Supplementary material, Delphi round 1.

Figure S2. Agreement and disagreement with our proposed items in the Delphi survey round 2

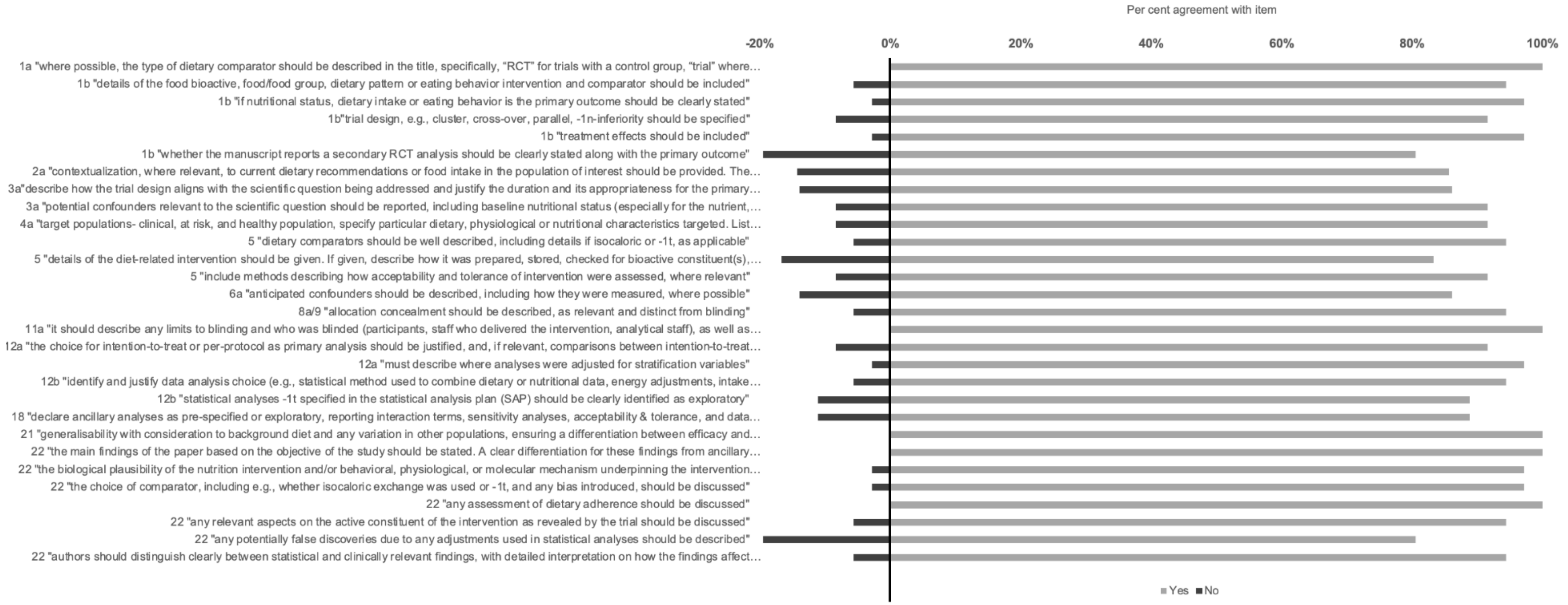

Figure legend: Agreement with the statement had to be >80% for acceptance. Some items are truncated due to length; the full text for each item can be found in the Supplementary material, Delphi round 2.
